# A cluster randomised trial of a classroom communication resource program to change peer attitudes towards children who stutter among grade 7 students

**DOI:** 10.1186/s13063-018-3043-3

**Published:** 2018-11-29

**Authors:** Rizwana Mallick, Harsha Kathard, A. S. M. Borhan, Mershen Pillay, Lehana Thabane

**Affiliations:** 1 University of Cape Town, Rondebosch, Cape Town, South Africa; 2 McMaster University, Hamilton, Canada; 3 University of KwaZulu Natal, Durban, South Africa

## Abstract

**Background:**

Classroom-based stuttering intervention addressing negative peer attitudes, perceptions, teasing and bullying of children who stutter (CWS) is required as part of holistic stuttering management because of its occurrence in primary school. This study was conducted in 2017, in 10 primary schools in the Western Cape, South Africa within lower (second and third) and higher (fourth and fifth) quintiles.

**Objectives:**

The primary objective of this study was to determine treatment effect at six months after intervention of grade 7 participants (Classroom Communication Resource [CCR] intervention versus no CCR) using global Stuttering Resource Outcomes Measure (SROM) scores in school clusters. The secondary objective was to determine grade 7 participant treatment effect on the SROM subscales including Positive Social Distance (PSD), Social Pressure (SP) and Verbal Interaction (VI). The subgroup objective was to determine any difference in the primary outcome between schools between and across quintile clusters (lower and higher).

**Methods:**

Once schools were stratified into lower and higher quintile (which are defined according to geographical location, fee per school and resources) subgroup clusters, schools were assigned randomly to control and intervention groups consisting of grade 7 participants who were typically aged ≥ 11 years. Teachers received 1 h of training before administering the single-dose CCR intervention over a 60–90-min session. The CCR intervention included a social story, role-play and discussion. All participants viewed a video of a CWS and stuttering was defined at baseline. The SROM measured peer attitudes at six months after intervention. Randomisation was stratified by quintile group using a 1:1 allocation ratio. Full blinding was not possible; however, the outcome assessor was partially blinded and the analyst was also blinded. Generalised estimating equations (GEE) was used assuming an exchangeable correlation structure to analyse the data adopting an intention-to-treat principle. Multiple imputation was used to handle missing data. Criterion for statistical significance was set at alpha = 0.05.

**Results:**

Ten schools were randomly allocated to control (k = 5) and intervention groups (k = 5), with *n* = 223 participants allocated to intervention and *n* = 231 to control groups. A total of 454 participants completed the SROMs in control (*n* = 231) and intervention (*n* = 223) groups and were analysed at baseline and six months after intervention. There was no statistically significant difference on the global SROM score (mean difference − 0.11; 95% confidence interval [CI] − 1.56–1.34; *p* = 0.88). There were also no significant differences on SROM subscales: PSD (mean difference 1.04; 95% CI − 1.02–311; *p* = 0.32), SP (mean difference − 0.45; 95% CI − 1.22–0.26; *p* = 0.21) and VI (mean difference 0.05; 95% CI − 1.01–1.11; *p* = 0.93). Additionally, there was no significant subgroup effect on the global SROM score (lower versus higher quintile subgroups) (interaction *p* value = 0.52). No harms were noted or reported.

**Conclusion:**

No statistically significant differences were noted. It is possible that the time frame was too short to note changes in peer attitudes and that further study is required to confirm the findings of this study.

**Trial registration:**

Clinicaltrials.gov, NCT03111524. Registered on 9 March 2017.

## Background

Culture, climate, ethos [1] and the ecological school system [2] may influence perceived scholastic experience with consequences on social and academic performance and functioning. These experiences vary within schools and classrooms. For example, school culture may be toxic for children who experience teasing, bullying, depression, reduced social and academic interactions [3, 4] and social rejection [5]. Teasing, bullying and general unacceptable behaviour at school, as listed above, have been studied extensively within the school context due to the grave consequences children face due to negative peer interactions [1–5]. Factors including age, race, learning difficulties, disabilities and health status are reported as predictive factors of teasing and bullying [2] including stuttering. Given the reported literature, the focus of this study is on peer attitudes, teasing and bullying, while using stuttering as an example of a vulnerable population.

The consequences reported above are commonly reported by children who have negative experiences of stuttering at school, caused by negative perceptions [3, 4], attitudes and interactions between children who stutter (CWS) and their peers [3, 6–9]. Grade 7 children are found to be most vulnerable and susceptible to teasing and bullying due to emotional changes that occur at this age [5]. It is for this reason that peer attitude and attitude change be addressed within the school context because of the damaging effects of negative peer attitudes on peer perceptions towards CWS [5–8]. Furthermore, it is reported that the basis of attitudes, formed by beliefs and knowledge, may be a predictive factor in behaviour [10]. It is, therefore, understood that there is a possibility of improving attitudes if underlying beliefs are targeted [10]. This is possible while acknowledging the complex and multifaceted link between attitudes, attitude change and behaviour change [11, 12]. For this reason, this study placed focus on attitude as the precursor to behaviour change [13] using a stuttering intervention tool while behaviour change itself was not studied.

Traditionally, CWS often receive individualised speech therapy sessions that target speech fluency with a focus on reducing core and secondary behaviours of the stutter. However, this study is concerned with another dimension of stuttering intervention. It focuses on group interventions for peers of CWS with specific emphasis on environmental factors. The focus on environmental factors are considered integral as part of holistic stuttering intervention that is guided by the International Classification and Functioning of Disability (ICF) framework [14]. Classroom-based stuttering interventions are recommended because CWS and their peers spend the majority of their day together with their teachers [15], supporting this study of peer attitudes within school clusters. It is also recommended that classroom-based interventions may empower teachers in the South African (SA) context who requested help to address negative attitudes towards stuttering in the classroom [16]. Classroom-based stuttering interventions to address peer attitudes, teasing and bullying are thus encouraged within the school context [14, 17, 18] as part of a robust stuttering intervention. To date, the Teasing and Bullying: Unacceptable Behaviour (TAB), a teacher-administered classroom-based intervention consisting of classroom activities to address peer attitudes towards CWS over a period of a few weeks and lessons, showed positive results in Canada when managing peer attitudes towards stuttering. Due to the TAB not being appropriate for SA, given its contextual needs (language, culture, time and technological differences), it gave rise to a SA equivalent tool, the Classroom Communication Resource (CCR) intervention, the intervention of interest in this study.

### Objectives

The primary objective was to determine treatment effect of grade 7 participants of the CCR intervention versus no CCR using the global SROM score at six months after intervention in school clusters. The secondary objective was to determine grade 7 participant treatment effect on the SROM subscales Positive Social Distance (PSD), Social Pressure (SP) and Verbal Interaction (VI). The subgroup objective was to determine any difference in effect on the primary outcome between quintile subgroups (lower and higher).

## Methods

The design and methods description for this trial is described below, while further details can be found in Mallick et al. [19].

### Trial design

A stratified cluster randomised controlled trial (RCT) was conducted using a 1:1 allocation ratio whereby schools were the unit of randomisation and were stratified into two quintile groups (lower versus higher quintile groups). No changes were made to the methods after the trial commenced as a previous pilot study [20] guided this study.

### Participants and schools: eligibility criteria and study setting

Participants were in grade 7, aged ≥ 11 years, and attended public lower and higher quintile schools with English as the language of learning and teaching within the Western Cape (WC) Metro urban area. Both lower and higher quintile schools were included to ensure a representative school sample of the WC considering the continued socio-political resource and funding disparities that form part of its current reality [21, 22]. A variety of schools, as stipulated by quintile classification, were additionally included given that experiences and peer attitudes may differ according to school and quintile groups.

### Intervention

Teachers were trained over a 1-h session and given a two-week period to review and prepare for the administration of the single-dose CCR intervention. Teachers administered the intervention over a 60–90-min lesson. Teachers read the social story, participants acted out the role-play and teachers facilitated a discussion around communication and communication difficulties, teasing and bullying, acceptance, diversity and difference. Observational notes were made by the researcher and assistants while the CCR was administered.

### Control

Usual care was followed, i.e. no activities described in the intervention were completed in control groups.

### Outcome measure

The primary outcomes measure used in this study was the SROM. The primary endpoint was the global SROM evaluated at six months after intervention. The secondary outcomes were the SROM subscale scores at six months after intervention. The SROM was developed for SA, based on the Peer Attitude Towards Children who Stutter (PATCS) which met its criterion reliability [15, 18]. The SROM was also psychometrically tested and found to be valid and reliable [5, 16, 23, 24]. The repeated use of the SROM in this study is acknowledged; however, there is no other available equivalent validated outcomes measure. Furthermore, the time lapse between baseline and six-month post-intervention measures may have reduced re-intervention bias. No changes were made once the trial commenced.

### Sample size

A sample size of *n =* 350 students (k = 10 schools) in the two groups was proposed while a sample size of *n* = 454 was included in this study (k = 10 schools). This study aimed to yield a statistically significant result with 80% power at alpha = 0.05, assuming an intention-to-treat (ITT) principle for analysis. A generalised estimating equations (GEE) model was implemented using six-month post-intervention (adjusting from baseline) mean global SROM scores. Sample size and computation was guided by previous studies [16, 20, 25, 26] assuming normally distributed global SROM scores with a mean difference of 5.25 (77.91 intervention group and 72.66 control group), ICC (intra-school correlation coefficient) of 72.70 and a common within-group standard deviation of 11.90.

### Randomisation

#### Allocation: sequence generation, allocation concealment mechanism and implementation

Following the collection of permission and consent outlined in the protocol paper of this study [19], schools in pre-existing clusters due to quintile classification (higher versus lower) were randomised. A once-off computerised allocation sequence was generated and placed in envelopes by a statistician with a 1:1 random allocation ratio and 2:1 randomisation stratification for quintiles. Schools were classified into two quintile groups (higher versus lower) based on geographical location, fee paid per learner and resources allocated to schools as determined by the country’s education department.

### Blinding

While blinding was not possible, some procedures were put into place to uphold the validity of this study. This included the primary researcher being partially blinded in terms of the capturing of the SROM and the use of a team of research assistants for all processes (SROM administration, as outcomes assessors for capturing and rechecking SROM responses as well as for teacher training and observations). The team ensured that those who administered the SROMs did not capture the data and those who captured data did not recheck the same SROMs. All outcomes assessors (research assistants) who captured and rechecked data were blinded as each SROM was coded. The analyst was also blinded.

### Statistical methods

The grade 7 participant was the unit of analysis. The ITT principle was followed and the GEE method was used to compare groups and subscales of the global SROM, which accounted for clustering within schools assuming a within-school exchangeable correlation structure. Results were reported as an estimate of difference between groups, as per the Consolidated Standards of Reporting Trials (CONSORT) Statement, along with a 95% confidence interval (CI) and associated *p* value (three decimal places). Multiple imputation was also used to impute missing data and five datasets were generated. Moreover, subgroup analysis was performed and analysis of constructs, determined by an interaction term (e.g. quintile group [lower versus higher] × intervention [CCR versus usual practice]). Subgroup analysis is supported by a previous study which showed schools behaving as clusters and as quintiles [20], while no large-scale findings have been reported. Treatment effect was additionally analysed in terms of the direction and magnitude.

## Results

### Recruitment

**Fig. 1:**
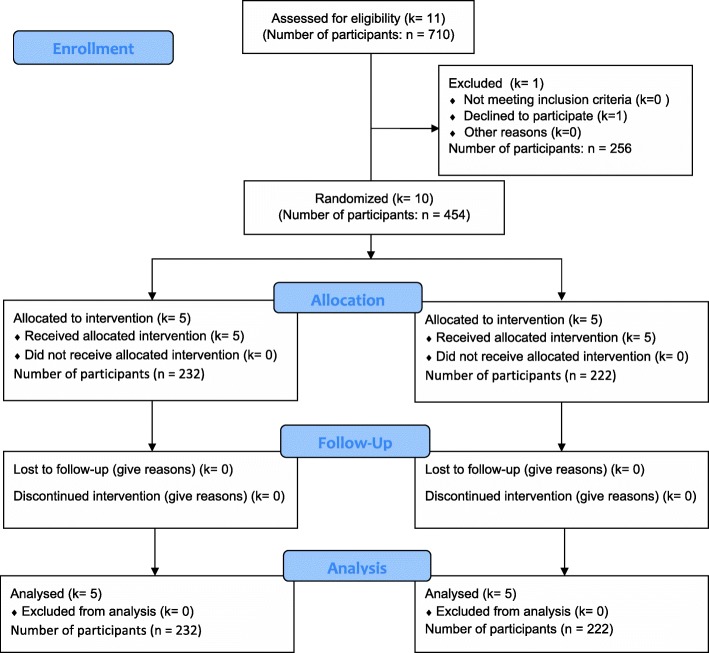
CONSORT 2010 flow diagram

Ten schools were recruited for adequate participant enrolment, as determined and described in the protocol paper [19]. Recruitment commenced on 19 January 2017, baseline data were collected on 6–13 February 2017 and all follow-up data on 1–6 August 2017 when the trial closed. See Fig. 1 for the CONSORT diagram showing flow of schools and participants in the trial.

### Baseline data and numbers analysed

The total sample analysed was *n* = 454 with *n* = 223 in the intervention group and *n* = 231 in the control group with 42% and 43% boys in each group, respectively. There were five clusters (schools) in each group (intervention and control) while six clusters were from the higher quintile and four from the lower quintile. Table 1 provides a summary of the baseline characteristics by intervention and control group.

**Table 1:** Baseline characteristics of study participants by intervention and control group

	Intervention (*n* = 223)	Control (*n* = 231)
Clusters (*n*)	5	5
Cluster size (*n*)		
Mean (range)	45 (30–54)	46 (18–68)
Quintile (*n* (%))		
Higher	115 (52)	118 (51)
Lower	108 (48)	113 (49)
Gender (*n* (%))		
Male	95 (43)	96 (42)
Age (years)		
Mean (SD)	13 (0.76)	13 (0.84)
Baseline score		
Mean (SD)		
SROM	60.55 (7.13)	60.67 (7.86)
PSD	39.31 (7.62)	39.71 (8.44)
SP	12.22 (3.54)	12.20 (3.37)
VI	9.02 (3.22)	8.77 (3.19)

### Outcomes and estimation: primary (ITT) analysis

**Fig. 2:**
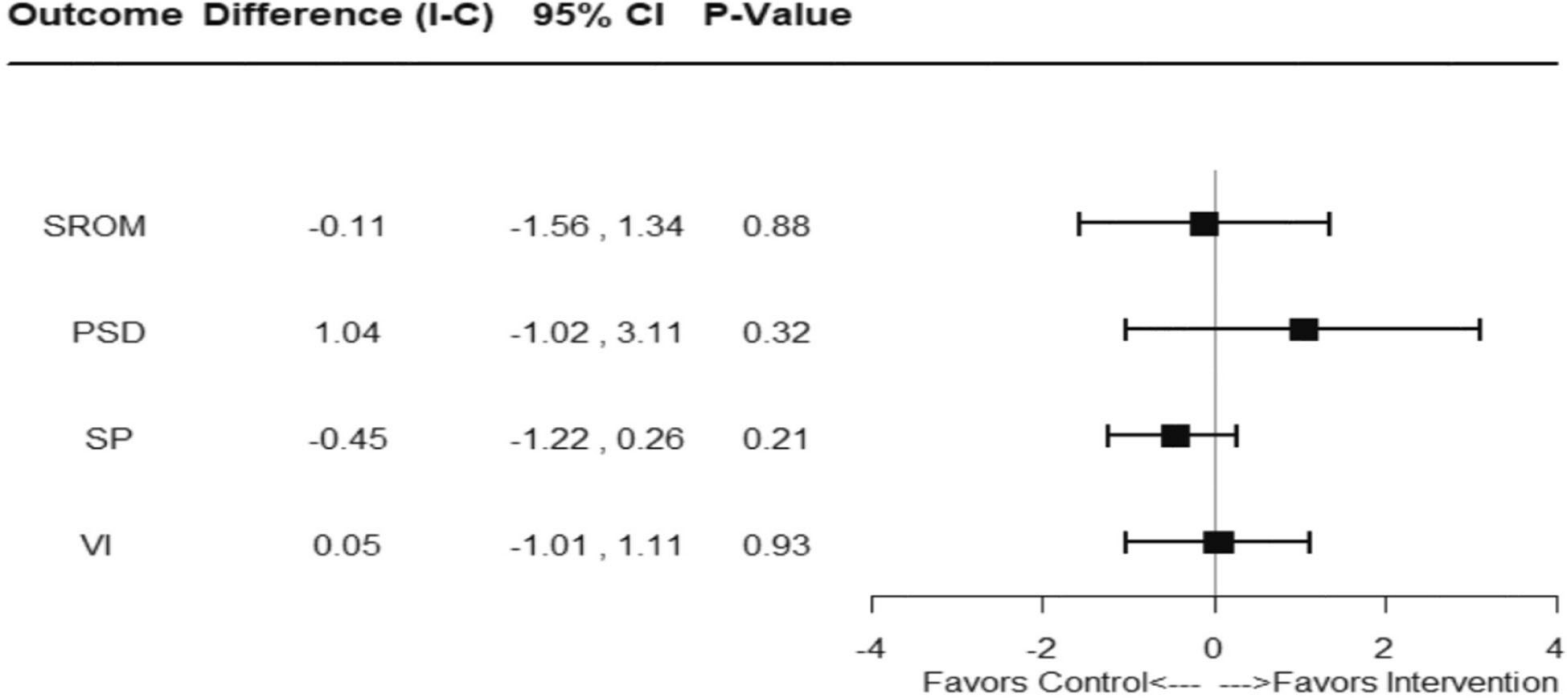
*Forest plot graph* showing difference (I-C), 95% confidence intervals and *p* values for the SROM and its constructs PSD, SP and VI at six months after intervention using different outcomes for ITT analysis

Figure 2 shows no statistically significant differences on the global SROM score (mean difference − 0.11; 95% CI − 1.56–1.34; *p* = 0.88). Similarly, no statistically significant differences were observed in the constructs of PSD (mean difference 1.04; 95% CI − 1.02–3.11; *p =* 0.32), SP (mean difference − 0.45; 95% CI − 1.22–0.26; *p* = 0.21) and VI (mean difference 0.05; 95% CI − 1.01–1.11; *p* = 0.93). The results remained robust when sensitivity analysis was conducted using complete case, per-protocol analysis.

### Ancillary analyses: subgroup analysis

**Fig. 3:**
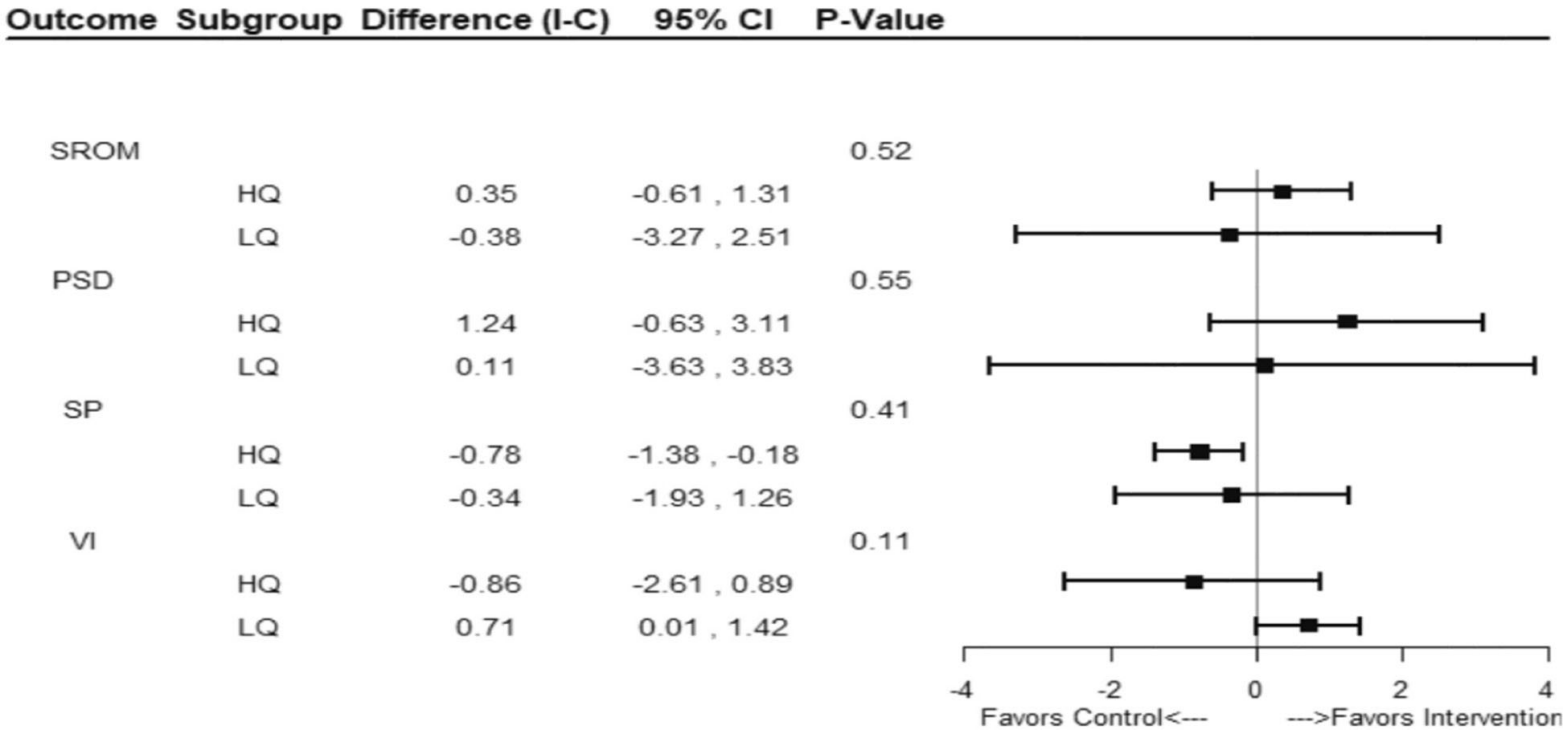
*Forest plot graph* showing difference (I-C), 95% confidence intervals and *p* values for the SROM and its constructs PSD, SP and VI at six months after intervention in quintile subgroups using different outcomes for ITT analysis

Subgroup analysis was pre-specified as a previous study showed that participants behaved as clusters [19]. The subgroup analysis findings showed no statistically significant difference of the SROM subscales where no significant subgroup effect on the global SROM score of lower versus higher quintile subgroups was noted with an interaction *p* value of 0.52. No statistically significant differences were noted within the constructs of PSD scores (interaction *p* value 0.55), SP (interaction *p* value 0.41) and VI (interaction *p* value 0.11). Sensitivity analysis based on per-protocol approach showed similar results (Fig. 3).

### Harms

Teachers were consulted for this study before baseline data collection began to identify CWS in the class to determine if they wished for the study to commence in their class. No concerns or need for counselling was reported.

## Discussion

### Limitations

A key limitation to this study is that there is no published information about the psychometric properties of the SROM. The only information available regarding the validity and reliability of the SROM has been cited in Mallick et al. [19]. Given the findings of this study, it is felt that further studies are required to consider SROM sensitivity and explore other potential tools due to the complexity of studying attitudes [12]. As described in the discussion, time frame and contextual difficulty were also limitations to this study.

### Generalisability

The generalisability of this trial should be interpreted with caution. This trial provides valuable findings for the WC lower and higher quintile schools but may not accurately reflect other provinces in SA.

### Interpretation

Overall, there were no statistically significant differences noted on the global SROM as well as within the constructs (PSD, SP, VI); however, on subscales, the results seemed consistent. The results, however, showed that the direction of change of the treatment effect was consistent with the hypothesis, with a difference of as large as 3.30—which is the upper limit of the 95% CI for the difference in the global SROM score. There were additionally no statistically significant differences in quintile subgroups and sensitivity analysis showed similar results with ITT. Additionally, there were no significant subgroup differences. However, the direction of the treatment effect was consistent with the hypothesis. Despite contextual discrepancies in the schools included in this study, the CCR intervention showed that the lower and higher quintile schools behaved similarly. This result is interesting given the contextual complexities of schools in the WC and the use of quintile classification. It signals that the CCR intervention may be useful for both quintiles in future planning related to this study and possibly to guide future teasing and bullying related school policy.

A cluster RCT conducted with grade 7 learners in peer attitudes towards disability in France noted that the study of cognitive, affective and behavioural components is important when measuring peer attitudes [27]. This finding supports the use of the SROM which includes these components within the constructs of PSD, SP and VI. Despite the inclusion of these components, it is possible that the SROM is not sensitive enough to pick up changes in attitudes. However, in the absence of another outcomes measure, the SROM was used given the available data on the reliability and validity testing so far [16]. A similar study targeting classroom-based peer attitudes through teacher administered activities and videos for peers with Tourette’s syndrome also used the condition as an example to improve general attitudes and beliefs towards disabling conditions [10], much like the CCR intervention. The study of peer attitudes towards Tourette’s syndrome recommended that baseline and post-intervention findings yield important results but that classroom observations should also be considered because of the changing nature of attitudes over time, i.e. to see if any attitudinal changes persist over time [10] and manifest beyond that of the outcomes measure.

In addition to the potential reduced sensitivity of the SROM to measure peer attitudes, it should also be considered that attitude and attitude change is cited as being a complex topic to study [11, 12]. In fact, it is reported that quantifying, measuring and exploring attitude is challenging and that there is no one way of doing so [12]. It is particularly complex to measure attitude knowing that the formation of attitude and attitude change changes over time because attitude is learnt [28] as a continuous process [29]. As such, it is possible that six months was too short to allow for attitudinal change to be measured. Perhaps this CCR intervention study should consider two things: (1) thoughts and beliefs cannot solely be measured using questionnaires and so additional observational data may be needed; and (2) there is no one optimal time frame in which attitude can be measured. It is important that when complex topics (such as peer attitude) are being studied, other measures and methods of commenting on the effectiveness of interventions are drawn upon without solely relying on statistically significant results.

Another crucial factor to consider is the time frame challenges that were experienced in this study due to the context of school-based research. First, school-based research comes with its own challenges such as time constraints that research imposes on schools as well as those stipulated by the Western Education Department (WCED), schools and teachers. Given the busy academic schedule, research can only be conducted when and where the school is able to accommodate the research. As mentioned, the WCED has strict regulations around when school-based research may take place and as such it was stipulated that the research could only be conducted between 1 February and 29 September 2017. These dates, however, do not consider school holidays, other school-specific commitments and the time needed to set up research. The tasks needed to set up the research required careful planning and included recruitment, obtaining permission, consent and assent from schools, principals, teachers, parents and participants, as well as arranging suitable dates and times for baseline data, teacher training, administration of the CCR and follow-up dates for the six months of post-intervention data. As illustrated by the details of the logistics of the research processes in schools, this meant that the data were unable to be collected over the eight-month period as stipulated by the WCED. As a result, the six-month period after the administration of the CCR intervention was the longest possible time frame that could be made available for this research. The research could also not be extended as the participants moved to the next grade and or school in January 2018 and thus there could be no way of including all participants beyond September 2017. In conclusion, this study could therefore be improved by replicating its findings, further study of the CCR intervention, publishing the findings around the sensitivity and psychometric properties of the SROM and considering additional other measurement tools such as observations.

## Conclusions

There were no statistically significant differences on the global score and subscales of SROM. It is possible that this may be due to the short time frame to detect changes in peer attitudes. It is therefore required that further studies be conducted to confirm the findings of this study. This study is the first speech-language therapy (SLT) cluster stratified RCT specifically in teasing and bullying classroom-based stuttering intervention for peers of CWS in SA. This study has thus shown that a RCT is feasible, despite challenges of conducting school-based research. For this reason, it is felt that other RCTs are needed to replicate the results of this trial in different settings (not just in the WC in SA) subject to rigorous processes. Replicating this study would be instrumental in producing more substantive findings to guide policy when working with the WCED. There is also a need for further RCTs, given that teachers have requested assistance with managing stuttering and communication in the classroom. Through replicating the findings, evidence-based practices may be developed which may inform SLT practices in the future.

### Protocol

The protocol was published in 2018 and may be accessed on the *Trials* journal.

## Abbreviations

CCR: Classroom Communication Resource
CWS: Children who stutter
GEE: Generalised estimating equations
ICC: Intra-school correlation coefficient
ICF: International Classification and Functioning of Disability framework
ITT: Intention-to-treat
NRF: National Research Foundation
PATCS: Peer attitudes towards children who stutter
PERC: Programme for Enhancement of Research Capacities
PRS: Protocol registrations and results system
PSD: Positive Social Distance
RCT: Randomised controlled trial
RISA: Research and innovation support and advancement
SA: South Africa
SLT: Speech-language therapy
SP: Social Pressure
SROM: Stuttering Resource Outcomes Measure
TAB: Teasing and Bullying behaviour
VI: Verbal Interaction
WC: Western Cape
WCED: Western Cape Education Department
